# Opioid Prescription Following Wrist and Ankle Fracture Fixation in Scotland—Tradition Prevails

**DOI:** 10.3390/jcm11020468

**Published:** 2022-01-17

**Authors:** William T. Gardner, David R. W. MacDonald, Matthew J. Kennedy, Alastair C. Faulkner, Joshua R. McIntyre, Patrice Forget, Stuart A. Aitken, Iain M. Stevenson, on behalf of the SCORE Collaborative

**Affiliations:** 1 Department of Trauma & Orthopedics, Aberdeen Royal Infirmary, Aberdeen AB25 2ZN, UK; david.macdonald7@nhs.scot (D.R.W.M.); iain.stevenson@nhs.scot (I.M.S.); 2 Department of Trauma & Orthopedics, Raigmore Hospital, Inverness IV2 3UJ, UK; 3 Department of Trauma & Orthopedics, Queen Elizabeth University Hospital, Glasgow G51 4TF, UK; mjkennedy92@gmail.com; 4 Department of Trauma & Orthopedics, Royal Alexandra Hospital, Paisley PA2 9PJ, UK; 5 Department of Trauma & Orthopedics, Ninewells Hospital, Dundee DD2 1SG, UK; alastair.faulkner@nhs.scot; 6 Department of Trauma & Orthopedics, Royal Infirmary of Edinburgh, Edinburgh EH16 4SA, UK; joshua.mcintyre@nhslothian.scot.nhs.uk; 7 Department of Anaesthesia, NHS Grampian, Aberdeen AB25 2ZD, UK; patrice.forget@abdn.ac.uk; 8 Epidemiology Group, Institute of Applied Health Sciences, School of Medicine, Medical Sciences and Nutrition, University of Aberdeen, Aberdeen AB25 2ZD, UK; 9 Department of Trauma & Orthopedics, MaineGeneral Hospital, Augusta, ME 04330, USA; saaitken@me.com

**Keywords:** trauma, wrist, ankle, opioid, analgesia

## Abstract

The American ‘opioid crisis’ is rapidly spreading internationally. Perioperative opioid use increases the risk of long-term opioid use. We review opioid use following wrist and ankle fracture fixation across Scotland, establishing prescribing patterns and associations with patient, injury, or perioperative factors. Six Scottish orthopedic units contributed. A total of 598 patients were included. Patient demographics were similar across all sites. There was variation in anesthetic practice, length of stay, and AO fracture type (*p* < 0.01). For wrist fractures, 85.6% of patients received a discharge opioid prescription; 5.0% contained a strong opioid. There was no significant variation across the six units in prescribing practice. For ankle fractures, 82.7% of patients received a discharge opioid prescription; 17% contained a strong opioid. Dundee and Edinburgh used more strong opioids; Inverness and Paisley gave the least opioids overall (*p* < 0.01). Younger patient age, location, and length of stay were independent predictors of increased prescription on binary regression. Despite variability in perioperative practices, discharge opioid analgesic prescription remains overwhelmingly consistent. We believe that the biggest influence lies with the prescriber-institutional ‘standard practice’. Education of these prescribing clinicians regarding the risk profile of opioids is key to reducing their use following surgery, thus lowering long-term opioid dependence.

## 1. Introduction

The USA and Canada have struggled with an ‘opioid crisis’ over the last few decades and have had to implement rigorous measures to monitor and regulate their prescription [1]. However, this issue is not unique to the North American continent; rates of opioid use and abuse are rising within the UK [2], with the issuing of prescriptions not always in line with best practice guidance [3,4].

There is evidence that perioperative opioid use increases the risk of long-term opioid use [5,6,7], but there is limited evidence as to the ‘correct’ amount of analgesia a patient requires following certain orthopedic operations [8]. As a result, it has been suggested that the rationale behind opioid prescribing is often more to do with accepted practices and prescriber-dependent behavior than any true clinical reason [9,10].

We hypothesized that opioid prescribing will be broadly similar across Scotland, with no links to any identifiable factor. Due to the high incidence of wrist and ankle trauma across the world, we chose to examine these injuries and their opioid use as a surrogate for opioid prescribing in the ‘ambulatory’ orthopedic population. Our primary outcome was to investigate new opioid prescriptions on discharge following surgical management of a wrist/ankle fracture. Secondary outcomes reviewed patient factors, injury, surgical and anesthetic variables that may influence post-operative prescribing patterns.

## 2. Materials and Methods

The study was conducted across six orthopedic trauma units under the Scottish Orthopedic Research Collaborative (SCORE), encompassing five health boards and four major trauma centers. All regions are involved in the post-graduate training of doctors across Scotland. The hospitals included Aberdeen, Dundee, Edinburgh, Inverness, Paisley, and Glasgow. Local health board approval was sought for each region.

Inclusion criteria: >16 years of age with closed physes and isolated (the only injury location) fracture of their distal radius or ankle requiring surgical management in the form of plate fixation. Ankle fractures could be of any type, including bimalleolar and trimalleolar. Exclusion criteria: polytrauma, pediatric patients, injuries managed non-operatively, and injuries managed with surgical techniques other than plate fixation (external fixation, K-wire fixation, etc.). All included were operated on within the year 2020.

Patients were identified from theater records. Electronic health records were reviewed to retrieve demographic data including pre-injury opioid use and history of anxiety/depression (as coded in their past medical history), both previously shown to increase the risk of higher post-operative opioid requirements [11,12]. Surgical procedure (number of incisions), anesthetic type (general vs. regional), ASA grade (a marker of comorbidity), length of stay, and discharge prescription/opioid type (strong opioids, e.g., morphine and oxycodone, or weak opioids, e.g., dihydrocodeine and codeine) were also recorded. The hospital picture archiving and communication (PACS) system was used to determine the AO fracture type. Briefly for wrists, type A: extra-articular, not involving the joint surface; type B: partial articular, with the fracture involving one part of the articular surface and the remainder of the joint remaining attached to the metaphysis/diaphysis; type C: intra-articular, with the fracture disrupting the joint surface and completely separating it from the diaphysis. For ankles, type A: infrasyndesmotic, type B: trans-syndesmotic, type C: suprasyndesmotic, all with reference to the lateral malleolus +/− medial (A/B/C) or posterior (B/C) lesions. Mechanism of injury was graded high if it was more than a mechanical fall from standing height (irrespective of activity performed; for example, team sports would be low energy and motor vehicle accident high energy). All supervising surgeons judged the reduction of the fracture to be satisfactory, and there were no cases revised for malreduction during the study period.

Ankle fracture patients and distal radius fracture patients were analyzed as two distinct groups. The continuous variable ‘patient age’ is presented as the median and range. Categorical variables are presented as frequencies and percentages. Data analysis was performed using SPSS version 22 (SPSS Inc., Chicago, IL, USA), with *p* < 0.05 used as identifying significance.

A sample size was calculated to reach a precision of 10% in the estimation of the incidence of new opioid treatment as the primary outcome. According to Young et al., the expected incidence of opioid prescription may range between 40% and 90% for ankle fracture (depending on the country) and 30% and 85% for wrist fracture, with differences of more than 20% between countries [13]. In the current study, a sample size of 50 patients per center, with an expected frequency of new opioid treatment of 85%, would thus allow us to calculate 95% confidence intervals between 75% and 95% and to unmask differences beyond these 95% CIs with *p* < 0.05.

Descriptive analyses are the most important part of the study. Results are presented per individual center and in total.

The characteristics of each of the hospital cohorts were compared using the Kruskal–Wallis test for continuous variables (age) and ordinal variables (fracture type, length of stay) and the chi-square test for categorical variables (gender, history of anxiety/depression, pre-injury prescription opioid use, anesthetic type, surgical approaches used). Primary outcome analysis was performed by comparing observed and expected frequencies of the presence or absence of a post-operative opioid prescription between cohorts using the chi-square test.

Regression analysis was used to examine our secondary outcomes. We performed bivariate analysis to identify the association of independent variables with the dependent variable of interest (post-operative opioid prescription). The Mann–Whitney U test and the chi-square test were used for continuous and categorical variables, respectively.

Independent variables with *p* < 0.1 or better were selected for inclusion in the regression analysis. We selected a binary logistic regression model to analyze the ability of the independent variables to influence the dependent variable, accounting for confounding. This produced the Nagelkerke R square, which provides an approximation of the proportion of variation in the dependent variable that can be explained by the independent variables. In addition, the model output produces the exponentiation of the B coefficient, Exp(B), for each independent variable included. The Exp(B) is an odds ratio, representing the effect that a one-unit increase in the independent variable has upon the odds of producing the dependent outcome: each unit increase in “X” multiplies the odds of “Y outcome” by Exp(B).

## 3. Results

A total of 598 patients were included in this retrospective cohort study, all of whom had received surgical management of an isolated acute distal radius (298) or ankle (300) fracture.

Table 1 shows the distal radius fracture cohort demographics. Median age, gender, rates of depression/anxiety, and pre-injury opioid use were similar across the six sites. Aberdeen and Glasgow had increased rates of pre-injury chronic pain diagnoses (*p* = 0.023), and Glasgow had increased use of neuropathic agents pre-injury (*p* = 0.004). AO fracture type, length of stay, and anesthetic practice were variable across all six groups (*p* < 0.001). High-energy mechanisms of injury were rarer in Dundee and Edinburgh (*p* = 0.014), and only Aberdeen and Edinburgh used dual approaches for a select few (*p* = 0.003).

Table 2 shows the ankle fracture cohort demographics. No significant difference was observed in median age, gender, rates of depression/anxiety and chronic pain diagnoses, pre-injury neuropathic or opioid analgesic use, fracture type, and energy of injury mechanism. A significant difference was observed in anesthetic practice (*p* < 0.001), number of approaches used during surgery (*p* = 0.040), and length of stay (*p* < 0.001).

### 3.1. Opioid Prescribing Practice per Region

Aberdeen, Inverness, Edinburgh, and Glasgow favored dihydrocodeine 30 mg four times daily for pain (28-tablet packs). Dundee and Paisley favored co-codamol. Strong opioids favored across the nation were either additional oral morphine sulfate solution 10 mg/5 mL 2–4 hourly as required for pain, 100 mL bottle, or a small supply of immediate-release oxycodone 5 mg 2–4 hourly as required for pain (28 pack). No patients were discharged on new sustained-release opioids.

### 3.2. Distal Radius Fracture Cohort

Across the 298 patients, 255 (85.6%) patients received a new discharge opioid prescription, of which 240 (80.5%) patients received a weak opioid prescription, and 15 (5.0) received a strong opioid prescription (Table 3). Prescription rates did not vary significantly (*p* = 0.407). One patient from the Glasgow cohort had missing data.

Univariate analysis (Table 4) identified no single variable as a causative factor for opioid prescription, with only AO fracture type A approaching significance (*p* = 0.088).

The binary logistic regression model accounted for only 3% of the variability in post-operative opioid prescribing (Nagelkerke R square, *p* < 0.001) and included only fracture type as a statistically significant predictor. Using type C fractures as the reference, patients with a type A fracture were more likely to receive an opioid prescription (Exp[B] = 2.3, 95%CI = 1.0–5.2, *p* = 0.04).

### 3.3. Ankle Fracture Cohort

Across the 300 patients, 248 (82.7%) patients received a new discharge opioid prescription, of which 197 (65.7) were for a weak opioid, and 51 (17.0%) were for a strong opioid (Table 5). Rates and strength of prescription varied significantly, with Dundee and Edinburgh using significantly more strong opioids in addition to their weak opioids and Inverness and Paisley giving the least discharge opioid prescriptions overall.

Univariate analysis (Table 6) revealed younger age (*p* < 0.001), location (*p* = 0.018) and longer length of stay (*p* = 0.018) as predictors of receiving a discharge opioid prescription. Pre-injury neuropathic agent use approached significance (*p* = 0.060).

Using any opioid discharge prescription as the dependent variable, the binary logistic regression model accounted for 23% of the variability in post-operative opioid prescribing (Nagelkerke R square, *p* < 0.001) and correctly classified 82% of cases. It included patient age, patient location, and length of stay as statistically significant predictors. For every one-year increase in age, patients were 3% less likely to receive an opioid prescription (Exp[B] = 0.97, 95%CI = 0.94–0.98, *p* < 0.001). When compared with Edinburgh, patients in Inverness (Exp[B] = 0.17, 0.04–0.59, *p* = 0.01) were less likely to receive an opioid prescription. When compared with patients staying 1 day, same-day discharges (i.e., 0 days) were less likely to receive an opioid (Exp[B] = 0.14, 0.04–0.51, *p* = 0.003). Patients staying 2 or more days were also less likely to receive a discharge opioid prescription than those staying 1 day (Exp[B] = 0.25, 0.08–0.66, *p* = 0.009).

Using a strong opioid discharge prescription as the dependent variable, the model accounted for 26% of the variability in strong opioid prescribing (Nagelkerke R square, *p* < 0.001), and correctly classified 83% of cases. It included patient location, pre-injury use of neuropathic medications, and length of stay as significant predictors. When compared with Aberdeen, patients in Dundee (Exp[B] = 26.7, 5.1–467.3, *p* = 0.002) and Edinburgh (Exp[B] = 53.2, 9.3–565.0, *p* < 0.001) were more likely to receive a strong opioid. Pre-injury use of neuropathic analgesic agents increased the odds of a strong opioid script by a magnitude of 4.3 (Exp[B], 95% CI = 1.4–10.6, *p* = 0.007). When compared with patients staying 1 day, same-day discharges (i.e., 0 days) were less likely to receive an opioid (Exp[B] = 0.16, 0.03–0.63, *p* = 0.02). Including the use of regional blocks had no effect on the models.

## 4. Discussion

This study shows significant variability in the patients, injuries, and perioperative practices when considering those that undergo distal radius fracture fixation across Scotland. Despite this, opioid analgesic prescription on discharge remains very consistent, with 85% of patients receiving new opioids. Of the patients in the distal radius cohort, 80% receive new weak opioids on discharge. Strong opioid prescription on discharge following a distal radius fracture was infrequent.

When considering the ankle fracture cohort, patient demographics and injury selection for surgical intervention are generally consistent across Scotland, but anesthetic and surgical practices do show some variation, along with length of stay post-operatively. Strength of opioids prescribed on discharge varied significantly, with Dundee and Edinburgh giving additional strong opioids as required on top of weak opioids and Inverness using significantly fewer opioids overall. These centers did not otherwise vary significantly in the patient and operative characteristics within their cohorts.

In the United States of America, the term ‘opioid crisis’ has been coined, with work being done to monitor and regulate opioid prescription. There is increasing concern that this crisis is spreading to Europe [1]. American research identified the three most important prescription factors influencing long-term opioid use as the use of modified-release opioids, repeat prescription following discharge, and the size of the initial prescription given [14,15]. Within the UK, as evidenced by our results, we favor weak opioids such as dihydrocodeine/co-codamol, and the rate of their use (and therefore risk of misuse) has increased significantly over the last decade [2,16]. Although our patients are supplied with a relatively small quantity of opioids following their operation by the hospital, the recent meta-analysis by Lawal et al. (2020) [11] involving over 1.9 million patients showed that 7% of patients continue to request opioid analgesia prescriptions more than 3 months after surgery, which if applied to our cohort alone is 41 patients—a noteworthy proportion, even if the generalizability of this remains debatable.

Scottish wrist fracture discharge opioid practices are broadly similar across the country. AO fracture type A showed a significant relationship with increasing opioid prescription, a finding not easily explained after controlling for the influence of other variables.

When examining the ankle cohort, younger age and pre-injury use of neuropathic agents showed an association with opioid discharge prescription. Length of stay was also a factor, with patients staying 1 night only receiving the most opioid prescriptions on discharge, although this may be linked to perception from the prescriber. If the patient goes home the same day, then they may be perceived to be ‘less sore’, and those staying multiple days being weaned off their opioids, or alternatively, being the older, frailer subgroup. An ambulating patient, remaining opioid naïve until discharge, may be seen as less suitable for opioids due to them being more at risk of toxicity or less at risk of needing them.

None of the variables examined in the wrist cohort except for AO type A fractures appear to have any individual effect on discharge opioid prescription rates, especially relevant considering the variation in practice across the six units studied. Other factors must be influencing opioid prescribing, namely prescriber culture and institutional practices. This has been researched previously [17,18,19], predominantly in the USA. Education of doctors-in-training is key to highlighting the dangers of opioid prescription, which might reduce the widespread practice of using opioids as the mainstay of post-operative pain control. Patient perceptions of pain and nociception may play a role; managing the pain expectations of patients post-operatively has the potential to reduce the perceived requirement for opioids [20].

Daliya et al. [3] performed a similar study within the UK, reviewing prescribing patterns on discharge following elective general surgical procedures. Their study highlighted a lack of guidance and education amongst medical staff, with a requirement for improved opioid stewardship at individual institutional levels. Our study suggests a similar issue within the orthopedic trauma setting through the prescribing practices currently occurring.

Higgins et al. [21] examined iatrogenic opioid dependence in patients prescribed opioids for chronic pain and reported a rate of 4.7%. They identify opioids within the weak category as being associated with a higher risk of long-term dependence/abuse, a concern when considering the discharge prescriptions within our trauma population.

Our regression models identified that the included variables accounted for 3% (wrists) and 23% (ankles) of variation, respectively. This supports our theory that other factors, namely institutional ‘standard practice’, are involved. We only included injuries managed surgically using standard internal fixation techniques, excluding external fixation, in order to try and standardize our patient cohort to ambulant patients with high-incidence injuries not requiring subspecialty interventions. Although we did not include all socioeconomic factors, we attempted to cover anxiety/depression diagnoses and pre-injury opioid use, two of the main factors shown to most influence the prescription of opioids post-operatively [20,22]. We have also not considered perioperative and inpatient opioid prescription [23,24]. The ongoing coronavirus pandemic will have inevitably influenced anesthetic practices [25]; however, this does not appear to have influenced discharge prescriptions. For this study, we have no data on analgesic consumption or any additional prescriptions provided in primary care due to the lack of crossover between primary and secondary care records in the UK.

## 5. Conclusions

This Scottish national multicenter study examining opioid analgesia prescription following wrist or ankle fracture surgery suggests the biggest influence on prescription practices lies with the prescriber rather than the patient. There are some links with younger age, pre-injury neuropathic use, and length of stay, but it is impossible to ignore the overwhelming influence of institutional ‘standard practice’.

Education of healthcare staff and patients is key to reducing the use of opioids following surgery, thus lowering the risk of their long-term use. Further prospective research will look to understand patient experiences and outcomes with respect to the analgesics they were provided with, to accurately stratify and validate analgesic practices in a patient-specific way.

## Figures and Tables

**Table 1:** Distal radius fracture cohort demographics.

Distal Radius	Aberdeen (50)	Dundee (49)	Edinburgh (49)	Inverness (50)	Paisley (49)	Glasgow (51)	*p* Value
Age, median years (IQr)	50 (38–62)	56 (52–60)	48 (34–62)	53 (48–59)	56 (49–63)	51 (38–63)	0.613 Kruskal–Wallis
Gender, *n* (%)							
Female	33 (66)	42 (86)	34 (69)	42 (84)	39 (80)	37 (73)	0.112
Male	17 (34)	7 (14)	15 (31)	8 (16)	10 (20)	14 (27)	Chi square
Depression/anxiety, *n* (%) Yes	10 (20)	9 (18)	8 (16)	10 (20)	10 (20)	10 (20)	0.996 Chi square
Chronic pain, *n* (%) Yes	7 (14)	0	1 (2)	4 (8)	4 (8)	8 (16)	0.023 Chi square
Pre-injury neuropathic medication, *n* (%)	0	1 (2)	3 (6)	4 (8)	5 (10)	10 (20)	0.004 Chi square
Pre-injury opioid use, *n* (%)							
strong	0	2 (4)	1 (2)	0	1 (2)	1 (2)	0.274
weak	4 (8)	3 (6)	4 (8)	2 (4)	8 (16)	4 (8)	Chi square
Fracture type, *n* (%)							
A	8 (16)	29 (59)	20 (41)	30 (60)	18 (37)	34 (67)	*p* < 0.001
B	28 (56)	12 (25)	14 (29)	13 (26)	6 (12)	8 (16)	Chi square
C	14 (28)	8 (16)	15 (30)	7 (14)	25 (51)	9 (17)	
High energy, *n* (%) Yes	11 (22)	2 (4)	3 (6)	13 (26)	9 (18)	9 (18)	0.014 Chi square
Anesthesia used, *n* (%)							
General only	36 (78)	20 (41)	5 (10)	49 (98)	43 (88)	21 (41)	*p* < 0.001
Regional only	6 (12)	28 (57)	0	1 (2)	4 (8)	25 (49)	Chi square
General plus regional	5 (10)	0	44 (90)	0	2 (4)	5 (10)	
Missing value		1 (2)					
Surgical approaches, *n* (%)							
Single	45 (90)	49 (100)	47 (96)	50 (100)	49 (100)	51 (100)	*p* = 0.003
Dual	5 (10)	0	2 (4)	0	0	0	Chi square
Length of stay, days (%)							
Same day	25 (50)	33 (68)	41 (84)	18 (36)	11 (22)	20 (39)	*p* < 0.001
1 day	22 (44)	8 (16)	5 (10)	25 (50)	25 (51)	29 (57)	Chi square
2 or more days	3 (6)	8 (16)	3 (6)	7 (14)	13 (27)	2 (4)	

**Table 2:** Ankle fracture cohort demographics.

Ankles	Aberdeen (50)	Dundee (51)	Edinburgh (50)	Inverness (50)	Paisley (51)	Glasgow (48)	*p* Value
Age, median years (Interquartile Range)	46 (30–70)	56 (40–64)	53 (37–64.5)	57 (47–67)	50 (32–64)	48.5 (35–60.5)	0.219 Kruskal–Wallis
Gender, *n* (%)							
Female	25	34	35	32	37	31	0.235
Male	25	17	15	18	14	17	Chi square
Depression/anxiety, *n* (%)							
No	31	35	36	36	38	39	0.334
Yes	19	16	14	14	13	8	Chi square
Chronic pain, *n* (%)							
No	48	47	45	46	44	42	0.659
Yes	2	4	5	4	7	5	Chi square
Pre-injury neuropathic medication, *n* (%)							
No	46	47	49	46	43	42	0.279
Yes	4	4	1	4	8	5	Chi square
Pre-injury opioid use, *n* (%)							
Strong	46	45	42	45	43	44	0.698
Weak	4	6	8	5	8	4	Chi square
Fracture type, *n* (%)							
A	5	3	3	9	7	3	
B	31	38	26	29	29	34	*p* = 0.137
C	14	10	21	12	15	11	Chi square
High energy, *n* (%)	3	4	0	5	6	5	0.264 Chi square
Anesthesia used, *n* (%)							
General only	24	13	7	29	30	14	*p* < 0.001
Spinal only	24	25	11	18	8	28	Chi square
One of above, plus regional	2	12	32	3	11	5	
(Missing value)		1			2	1	
Surgical approaches, *n* (%)							
Single	21	28	18	23	23	32	*p* = 0.040
Dual	29	23	32	27	28	16	Chi square
Length of stay, days (%)							
Same day	6	8	17	1	1	5	*p* < 0.001
1 day	21	8	17	18	10	23	Chi square
2 or more days	23	35	16	31	40	20	

**Table 3:** Rates of post-operative opioid prescription in the distal radius fracture cohort.

	Aberdeen	Dundee	Edinburgh	Inverness	Paisley	Glasgow	*p* Value
Post-operative opioid prescription, *n* (%)							
Strong						0	
Weak	1	5	3	4	2	43	
None	45	35	40	38	39	7	*p* = 0.407
(Missing values)	4	9	6	8	8	1	Chi square

**Table 4:** Univariate analysis of wrist fracture cohort.

	No Opioid (N)	Opioid (N)	*p* Value
Age	Median = 60.5	Median = 60	0.750 Mann–Whitney U test
Location			
Aberdeen	4	46	0.738
Dundee	9	40	Chi square
Edinburgh	6	43	
Inverness	8	42	
Paisley	8	41	
Glasgow	7	43	
Gender			
Female	34	192	
Male	8	63	0.426
Depression/Anxiety Diagnoses			
No	37	203	
Yes	5	52	0.196
Chronic Pain Diagnosis			
No	39	235	0.875
Yes	3	20	
Pre-Injury Neuropathic Agent Use			
No	39	236	
Yes	3	19	0.944
Pre-Injury Opioid Use			
No	39	229	
Yes	3	26	0.537
Fracture Type			
A	13	125	
B	14	67	0.088
C	15	63	Near significance
Use of Regional Block			
No	24	154	
Yes	18	101	0.691

**Table 5:** Rates of post-operative opioid prescription in the ankle fracture cohort.

	Aberdeen	Dundee	Edinburgh	Inverness	Paisley	Glasgow	*p* Value
Post-operative opioid prescription, *n* (%)							
Strong	1	16	17	7	6	4	*p* < 0.001
Weak	42	29	29	27	33	37	Chi square
None	7	6	4	16	12	7	

**Table 6:** Univariate analysis of ankle fracture cohort.

	No Opioid (N)	Opioid (N)	*p* Value
Age	Median = 61.5	Median = 51	<0.001 Mann–Whitney U test
Location			0.018 Chi square
Aberdeen	7	43	
Dundee	6	45	
Edinburgh	4	46	
Inverness	16	34	
Paisley	12	39	
Glasgow	7	41	
Gender			
Female	34	160	0.905
Male	18	88	Chi square
Depression/Anxiety Diagnosis			
No	39	176	0.585
Yes	13	71	Chi square
Chronic Pain Diagnosis			
No	50	222	0.151
Yes	2	25	Chi square
Pre-Injury Neuropathic Agent Use			
No	44	229	0.060
Yes	8	18	Near significance
Pre-Injury Opioid Use			
No	48	217	0.326
Yes	4	31	Chi square
High Energy Mechanism of Injury			
No	2	21	0.255
Yes	50	227	Chi square
Fracture Type			
A	3	27	0.303
B	37	150	Chi square
C	12	71	
Use of Regional Block			
No	43	185	0.230
Yes	9	62	Chi square
Length of Stay			
0 days	7	31	
1 day	5	92	0.018
2 or more	40	125	Chi square

## Data Availability

The data presented in this study are available on request from the corresponding author. The data are not publicly available due to the details of the injuries being potentially patient identifiable.

## Supplementary Materials

The following are available online at https://www.mdpi.com/article/10.3390/jcm11020468/s1, Membership of the Score Collaborators.
