# Supplementary material for: Opioid Prescription Following Wrist and Ankle Fracture Fixation in Scotland—Tradition Prevails
Source: J Clin Med. 2022 Jan 17;11(2):468. doi: 10.3390/jcm11020468 (PMC8781195; doi:10.3390/jcm11020468)
Supplement: Supplementary file 1 [file jcm-11-00468-s001.zip › jcm-1496295-supplementary.pdf]

Supplementary Material

Score collaborators:

James Beastall [2], David Munn [3], Bilal Jamal [3], Luke Murphy [4], Jibu Joseph [4],Rehan Mackenzie [5], Clarissa Hocking [5], Ramaa Parulekar [5], Sheng Wei Chiam [5], Arpit Jariwala [5], Nicholas Clement [6]
